# Harnessing the Web: How E-Health and E-Health Literacy Impact Young Adults’ Perceptions of Online Health Information

**DOI:** 10.2196/med20.4327

**Published:** 2015-12-31

**Authors:** Rowena Briones

**Affiliations:** ^1^ Virginia Commonwealth University Richard T. Robertson School of Media & Culture Richmond, VA United States

**Keywords:** e-health literacy, young adults, online information seeking, online health information

## Abstract

**Background:**

The rise of technology has changed how people take control of their health, enabling individuals to choose to live healthier lives and make better treatment decisions. With this said, the Internet has emerged as the channel used by individuals for actively seeking or passively receiving health information.

**Objective:**

To explore how young adults assess the quality of health information, and how they construct meaning of online health information in general. Through 50 in-depth interviews, this study aims to examine how and why young adults turn to the Web for health information, and what strategies they employ to ensure that they are getting credible information.

**Methods:**

A total of 50 in-depth interviews were conducted with young adults to explore how they make meaning of online health information. Depending on the geographic area of the participant, the interview took place face-to-face at a location convenient for them, over Skype, or over the telephone and lasted on average 40 minutes. The interviews were transcribed verbatim, fully retaining the speech style of the moderator and the participants. Data were analyzed using techniques from the grounded theory approach, using a constant comparative method to allow for themes to emerge from the transcripts.

**Results:**

The participants shared several benefits to this mode of health information seeking, claiming that it made for more productive visits with doctors and made health information more readily accessible through a variety of different formats. Additionally, the participants demonstrated their e-health literacy levels by discussing how they assessed online health information, engaging in a series of strategies that encompassed different aspects of e-health literacy. Social media channels were brought up by the participants as relatively new tools that can be used to assist in the seeking, understanding, and sharing of health information. However, participants also cautioned about the use of social media in regards to its informal nature, warning users to evaluate sources accordingly and to use these channels as supplementary outlets of information for more traditional channels.

**Conclusions:**

The use of the Internet and technology for health purposes is a growing area for both scholarship and practice that has strong implications for health consumers, medical professionals, and communicators alike. The findings that emerged from this research demonstrated that the online space is an acceptable channel through which young adults can find and share information. However, in spite of the rising usage of social media by this particular group, the findings showed that they were hesitant and wary of the channel, not seeing it as a resource for health information but more of a channel for networking and entertainment. In spite of this, this study shows that the online health information seeking behaviors is an area that warrants further exploration.

## Introduction

### Background

The rise of technology has changed how people take control of their health, enabling individuals to choose to live healthier lives and make better treatment decisions [1]. According to the Pew Internet and American Life Project, 80 percent of adult Internet users have looked online for health information regarding a specific disease or treatment [2]. Kreps and Neuhauser [3] argue that a “communication revolution” is brewing in terms of delivering health care promotion and service through the use of new health information technologies.

With this said, the Internet has emerged as the channel used by individuals for actively seeking or passively receiving health information. The fluidity of the Internet has defined it as both an impersonal or personal channel [4]. If a person is searching for information on diabetes, for example, the Internet is impersonal and does not require person-to-person interaction with others. If that same person were participating in an eating disorders online support group, then the Internet becomes a personal channel for seeking health information. Some of the biggest advantages of using the Internet for disseminating health messages are its constant availability, its ability to provide useful information, and the fact that it offers anonymity to users [5].

One particular public that warrants attention among researchers is the young adult population. As the generation that grew up with the Internet, young adults realize its value for health information. A 2009 Pew survey found that 93 percent of young adults aged 18-29 are on the Internet, with 72 percent searching for specific health topics [6]. Therefore, the purpose of this study is to explore how young adults assess the quality of health information, and how they construct knowledge/make meaning [7] of online health information in general. Through a series of in-depth interviews, this study aims to examine *how* and *why* young adults turn to the Web for health information, and what strategies they employ to ensure that they are getting the most credible information possible.

### Literature Review

#### Defining eHealth

According to Eng [8], eHealth is defined as “the use of emerging information and communication technology, especially the Internet, to improve or enable health and health care” (p. 1). Though this is the most frequently cited definition in the field of communication, other definitions of eHealth have emerged in the extant literature. A systematic review of definitions by Oh et al [9] resulted in 51 unique definitions of eHealth proposed within the literature (For a select list of definitions, see Table 1).

**Table 1:** Definitions of eHealth (as cited in Oh et al [9]).

Source	Definition
Mitchell (1999)	A new term needed to describe the combined use of electronic communication and information technology in the health sector. The use in the health sector of digital data – transmitted, stored and retrieved electronically – for clinical, educational and administrative purposes, both at the local site and at a distance.
McLendon (2000)	eHealth refers to all forms of electronic healthcare delivered over the Internet, ranging from informational, educational and commercial “products” to direct services offered by professionals, non-professionals, businesses or consumers themselves. eHealth includes a wide variety of the clinical activities that have traditionally characterized telehealth, but delivered through the Internet. Simply stated, eHealth is making healthcare more efficient, while allowing patients and professionals to do the previously impossible.
Medical Business News (2000)	eHealth is a convergence between the Internet and the health care industry to provide consumers with a wide variety of information relating to the health care field
Oracle Corporation (2000)	Healthcare transactions, encounters, messaging, or care provision occurring electronically.
Deluca, Enmark (2000)	eHealth is the embryonic convergence of wide-reaching technologies like the Internet, computer telephony/interactive voice response, wireless communications, and direct access to healthcare providers, care management, education, and wellness.
Prelow (2000)	eHealth is the process of providing health care via electronic means, in particular over the Internet. It can include teaching, monitoring (eg, physiologic data), and interaction with health care providers, as well as interaction with other patients afflicted with the same conditions.
Baur, Deering & Hsu (2001)	The most broad term is eHealth, with refers to the use of electronic technologies in health, health care and public health. (...) The various functions of eHealth [are]: (...) reference (electronic publishing, catalogues, databases); self-help/self-care (online health information, support groups, health risk assessment, personal health records), Plan/provider convenience services (online scheduling, test and lab results, benefit summaries), Consultation and referral (doctor-patient or doctor-doctor consultation via telemedicine systems, remote readings of digital image and pathology samples), eHealth commerce (sales of health related product and services) [and] Public health services (automated data collection, data warehouses, online access to population survey data and registries, advance detection and warning systems for public health threats). (...) This chapter uses the term eHealth to refer to the broadest possible range of interactive technologies applied to health and health care.
Orlikoff & Totten (2001)	The use of the Internet and related information systems and technology in all aspects of health care.
Eysenbach (2001)	eHealth is an emerging field in the intersection of medical informatics, public health and business, referring to health services and information delivered or enhanced through the Internet and related technologies. In a broader sense, the term characterizes not only a technical development, but also a state-of-mind, a way of thinking, an attitude, and a commitment for networked, global thinking, to improve health care locally, regionally, and worldwide by using information and communication technology
Blake (2001)	The combined use of electronic communication and information technology in the health sector. It is important to note that eHealth is much more than business transactions. It encompasses everything from digital data transmission to purchase orders, lab reports, patient histories and insurance claims.
Robert J Wood Foundation (2001)	eHealth is the use of emerging information and communication technology, especially the Internet, to improve or enable health and health care.
Wysocki (2001)	eHealth refers to all forms of electronic healthcare delivered over the Internet, ranging from informational, educational and commercial “products” to direct services offered by professionals, non-professionals, businesses or consumers themselves
Health e-Technologies Initiative (2002)	The use of emerging interactive technologies (i.e., Internet, interactive TV, interactive voice response systems, kiosks, personal digital assistants, CD-ROMs, DVD-ROMs) to enable health improvement and health care services.
Kirshbaum (2002)	There are many different definitions of eHealth: Electronic connectivity vehicle for improving the efficiency and effectiveness of healthcare delivery Enabling consumers/patients to be better informed about their healthcare Enabling providers to deliver better care in more efficient ways
Wyatt and Liu (2002)	The use of internet technology by the public, health workers, and others to access health and lifestyle information, services and support; it encompasses *telemedicine, telecare*, etc.
Staudenmeir (2003)	Any use of the Internet or related technology to improve: the health and wellness of the population; the quality of healthcare services and outcomes; efficiencies in healthcare services or administration
COACH (2003)	The leveraging of the information and communication technology (ICT) to connect provider and patients and governments; to educate and inform health care professionals, managers and consumers; to stimulate innovation in care delivery and health system management; and, to improve our health care system.
RX2000 (2003)	eHealth signifies a concerted effort undertaken by some leaders in healthcare and hi-tech industries to harness the benefits available through convergence of the Internet and healthcare. Access, cost, quality and portability have been concerns in the health care arena. It's evident from many recent surveys that both health consumers and healthcare professionals are frustrated with the maze of health care delivery. Some, therefore, are turning to the Internet for answers and cost effective solutions.
WHO (2003)	eHealth is a new term used to describe the combined use of electronic communication and information technology in the health sector OR is the use, in the health sector, of digital data-transmitted, stored and retrieved electronically-for clinical, educational and administrative purposes, both at the local site and at a distance
Southwest Medical Group	eHealth is an emerging field focused on medical information and health care services delivered or enhanced through advanced Internet or related technologies. In a broader sense, the term extends the scope of health care beyond its conventional boundaries. Conceptually, eHealth enables patients to easily obtain medical related services online from health care providers
eHealth Technologies (2003)	The use of emerging information and communication technology, especially the Internet, to improve or enable health and healthcare thereby enabling stronger and more effective connections among patients, doctors, hospitals, payors, laboratories, pharmacies, and suppliers

#### eHealth Literacy

With more and more people taking control of their health to learn about a variety of conditions, diseases, and topics through the patient empowerment movement (du Pré, 2011; Schulz & Nakamoto, 2012) the fields of medicine and public health are starting to shift into a more consumer-focused practice. Individuals are seeking health information from a variety of different sources, including interpersonal interactions, television, print media and the Internet [4]. However, with 53 percent of adults having intermediate levels of health literacy [10], it has become evident that health literacy is an issue that warrants further attention, as national literacy levels in regard to health have been found to be low (Hay, 2010; Koh & Rudd, 2015; Torpy et al, 2011).

With this said, navigating the online sphere adds another dimension to health literacy, especially for young adults. Even though the majority of this population is competent in computer use and Internet searching, Hansen et al [11] found that their success in finding specific health information varied, due to frustration over the sheer volume of information available, as well as determining the credibility and accuracy of the information. Thus, with 80 percent of adult Internet users looking for health information online [2], it became imperative that a conceptualization of eHealth literacy was needed in order to determine exactly how to operationalize this measure. as a way to determine how individuals come to understand online health information.

**Figure 1:**
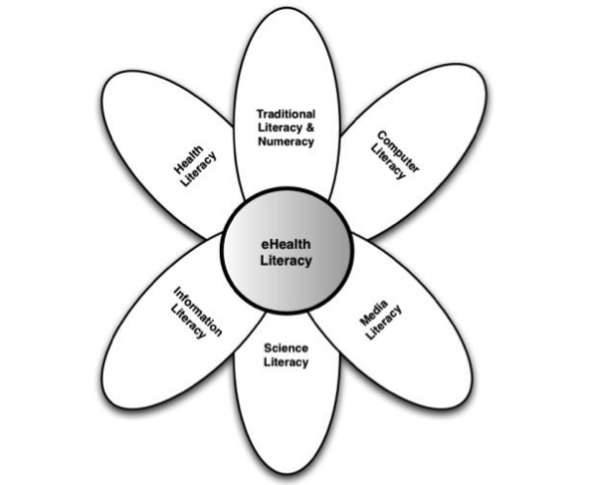
E-health literacy lily model.

There is currently one definition of eHealth literacy that is predominantly cited all throughout the literature, proposed by Norman and Skinner [12]: “The ability to seek, find, understand, and appraise health information from electronic sources and apply the knowledge gained to addressing or solving a health problem” (p. e9). Using the metaphor of the lily to model the idea of eHealth literacy, Norman and Skinner [12] claim that the “petals” of the lily consist of six core literacies that can be applied to the eHealth setting, with the “pistil” of eHealth literacy tying them all together (p. e9, see Figure 1). Norman and Skinner [13] have since designed the eHealth Literacy Scale (eHEALS) in order to assess health consumers’ perceived skills to better aid health educators and promoters in developing more personally relevant programs that more closely align with individuals’ levels of eHealth literacy. Additional research studies have further explored Norman and Skinner’s [12] conceptualization of eHealth literacy by way of testing eHEALS in various settings (e.g., Norman [14]; Xie [15]), with several scholars finding the scales to be easy-to-use and reliable (Brown & Dickson, 2010; [16,17]).

The six literacies are further broken down into two central types: analytic and context-specific. The analytic types of literacy include traditional literacy, media literacy, and information literacy. The analytic component encompasses skills that are applicable to a broad range of topics or contexts. The context-specific types of literacy include computer literacy, scientific literacy, and health literacy. As compared to its analytic counterpart, the context-specific component can only be contextualized and applied to a specific problem, circumstance, or situation.

##### Traditional Literacy

Based on the National Literacy Act of 1991, traditional literacy consists of “an individual’s ability to read, write and speak in English, and compute and solve problems at a level of proficiency necessary to function on the job and in society, to achieve one’s goals, and develop one’s knowledge and potential” [18]. Norman and Skinner [12] argue that in spite of the use of multimedia features such as still images, video and audio on the Web, they still contain a large text-based component that users need to understand in order to obtain eHealth resources.

Media Literacy

Norman and Skinner [12] describe media literacy as “a skill that enables people to place information in a social and political context and to consider issues such as the marketplace, audience relations, and how media forms in themselves shape the message that gets conveyed” (p. e9). In order to find online health information, users need to develop the cognitive and critical thinking skills necessary to truly assess and evaluate information online, especially with the sheer amount of information that is readily available online on a daily basis.

##### Information Literacy

The American Library Association [19] defines information literacy as “how knowledge is organized, how to find information, and how to use information in such a way that others can learn from them.” The information literate person would be able to locate the appropriate online resources to find information on a specific health topic, utilize the correct search strategies, and can filter through large amounts of information to find exactly what they need [12].

##### Computer Literacy

Logan’s [20] very simple and broad definition of computer literacy describes it as “the ability to use computers to solve problems.” Users need to consider the variety of options that are available in terms of computer technology, such as hardware and software, and be able to confidently utilize different computer systems in order to find health information. According to Norman and Skinner [12], a person could never become fully computer literate without quality access to computers and current information technology.

##### Scientific Literacy

A broad conceptualization of scientific literacy is an understanding of the nature, aims, methods, application, limitations, and politics of creating knowledge in a systematic manner [21]. Science literacy is a component of eHealth literacy because all health information is driven by science; users need to understand the process of how health information is scientifically discovered, and the various opportunities and limitations that come along with that scientific discovery [12].

##### Health Literacy

As previously discussed, health literacy consists of “the cognitive and social skills that determine the motivation and ability of individuals to gain access to, understand, and use information in ways that promote and maintain good health” [22]. Without the basic skills to successfully function in daily life and navigate the health system [23], users would never be able to translate that knowledge to the online space.

###  The Role of Social Media in eHealth

The growth and expansion of social media tools have provided yet another opportunity for eHealth campaign developers and educators. Social media can be seen as the “various electronic tools, technologies, and applications that facilitate interactive communication and content exchange” [24]. Platforms such as Facebook and Twitter are increasingly being used to faciliate a dialogue within the public health community, and to support a variety of different health issues such as suicide prevention and heart health [25]. What makes social media such a valuable tool is not necessarily its technological components, but the ability to create communities, spark health discussions, and provide interaction and engagement via the online space in real time [26].

A vital point for eHealth campaign developers to consider is the fact that social media should in no way replace traditional forms of communication, but rather should expand and enhance campaigns by way of creativity and broader reach [25]. In addition, like more traditional health communication campaigns, the same principles for effective campaign design still apply to a program utliizing social media channels, with similar principles as presented by Olgivy’s report “Using Social Media Platforms to Amplify Public Health Messages” [26]: (1) establish goals, objectives and strategies specifically for digital media; (2) identify audiences according to online information seeking, preferred social media networks, and social media usage; (3) optimize content by listening and engaging in bidirectional conversation on the specific health issue; and (4) evaluate digitally.

### Research Questions

RQ1: How do young adults assess the quality of online health information?

RQ2: How do young adults make meaning of online health information seeking in general?

## Methods

Qualitative methods were used to collect and analyze data. More specifically, 50 in-depth interviews were conducted with young adults to explore how they make meaning of online health information.

### Data Collection

To recruit participants, various university professors were contacted who provided access to students at different universities across the nation. Through these sampling methods a total of 50 participants were interviewed (for a listing of demographic information, see Table 2). Depending on the geographic area of the participant, the interview took place face-to-face at a location convenient for them, over Skype, or over the telephone and lasted on average 40 minutes. Participants were asked to sign a consent form prior to the start of the interview, and were asked to be audio recorded so that details could be gathered after the interview was conducted. . The interview protocol was reviewed and approved by the University’s Institutional Review Board (IRB). It followed a semi-structured format, which allowed the moderator the flexibility to change the order of the questions or clarify questions if needed [27]. Sample questions include the following: “Do you think the Internet is helpful in making decisions about your health?”; “How do you assess the quality and accuracy of online information?”; and “Do you use social media to get health information?”.

**Table 2:** Demographics of study participants.

Characteristic		Number of participants
**Sex**		
	Male	25
	Female	25
**Race**		
	White	30
	Black	3
	Hispanic	4
	Asian	13
**Age**		
	18-19	18
	20-21	12
	22-23	7
	24-25	13
**Sexual orientation**		
	Heterosexual	47
	Homosexual	3
**Relationship status**		
	Single	23
	In a relationship	27

### Data Analysis

The interviews were transcribed verbatim, fully retaining the speech style of the moderator and the participants. Memos and observer comments (OCs) were included throughout the transcripts to make note of interpretations of the interviewees and different questions or themes that may emerge from the data [28]. Data was analyzed using techniques from the grounded theory approach [29], using a constant comparative method to allow for themes to emerge from the transcripts [29-32]. First, open-coding procedures were used to examine the transcripts line-by-line to locate emerging themes and potential categories. Axial coding was then used to find how data can fit into the categorical themes that were identified in the first step, along with finding data that explicates the concepts presented in the proposed theoretical framework. According to Lindlof and Taylor [33], axial coding is a part of the integration process of the grounded theory approach that narrows down the number of categories by finding similarities across data in order to make the data clearer and more understandable. However, though single statements will be combined to create various concepts, stand-alone statements that were unique or exceptional were also coded, in an effort to avoid too much coherency in the data [34]. In addition, Corbin and Strauss [29] recommend using in-vivo codes in order to privilege the words of the participants, which refers to when a participant made a poignant point, these words were used verbatim as a code within the data analysis process. However, pseudonyms were used in the reporting of the results to protect the identity of the participants.

## Results

### RQ1: How Do Young Adults Assess the Quality of Online Health Information?

#### Assessing the Credibility of Online Sources

Several of the participants mentioned ways that they determined whether or not an online source is a credible, reliable piece of information. Amber claimed that sometimes some background research on the organization offering the information is needed:

I guess if you find out who posted the information on the website, and then if its a company that posted it, you can find out background research on that company. Its a lot of work, which is probably why I don’t do it, but I guess you just have to do the right research to figure out what the background of each person posting that information is.

For Diego, he felt that pages that are pushing to sell products are less likely to be trusted:

It’s more of an eye test. If it has motives, like trying to sell stuff with ads and has ulterior motives then no. And also there are a lot of sites that are backed by the government or societies or even some colleges, and I think that anything that is backed by a strong academic place is going to be more reliable.

Other participants, including Virginia, Erica, and Matt, want to see websites from reputable organizations, and were more likely to steer away from personal blogs. According to Erica,

Maybe because I sort of have a working knowledge in health and human services, but I feel like I trust sort of those bigger agencies like NIH and CDC. I don’t really trust something like if people have commented on something or a blog… I’m pretty skeptical on those things. I would trust something that had a more official feel to it.

Virginia also agreed, and said, “I think if they’re affiliated with a legitimate or I consider to be legitimate organizations like Red Cross or Greater Than AIDs for instance, I would take those to be more legitimate or more accurate than some random person’s blog site.” And Matt claimed he wanted “to see industry accreditation. If I see that its being sponsored by large recognizable associations of health I'm going to trust it more. I don't want to see a random medical blog where some “doctor” wrote [a post] from Kansas.”

#### Cross-Referencing/Cross-Checking Websites

Another way that participants assessed online health information was by going to a number of different websites and cross-referencing and cross-checking their information to see if they all match up. As Peter advised:

Don’t rely on one specific source for everything and that doesn’t even mean a form of media. If you’re going to get your health information through the Internet go around to a couple different websites. If you were going to a doctor and heard something you didn’t want to hear, you would want a second opinion. If you were looking up something online that doesn’t agree with you, it shouldn’t be a one-stop deal, it should be more of like what does this website say or what has this doctor researched about this topic.

Domenic claimed, “You have to be careful about what you see on there. I generally don't rely on one source. If one person says something, I'll check it on a different site. Anyone can post on the Internet. You need to correlate your results with other sources.” Matt had similar advice, and said, “There's a lot of good information out there but you can get easily sidetracked by mediocre, bad websites. It's a matter of making sure the info you find you pair with other credible websites in order to get a good, solid, general opinion on something. To make sure everything meshes.”

For Kyle, confirming information through other websites helps him assess to see if the original information is actually credible and correct:

So if something sounds right, then I think I'd assume it is, then I'd take the confirmation of other sites. So through looking at multiple unrelated sites that's how I assess the credibility. If something sounds wrong, I'm going to assume that it's probably wrong but also try to confirm that. Basically if something looks credible I'll assume it's correct if it's online. If it doesn't sound credible then I will also double check to see where this crazy information is coming from.

#### Addressing Website Characteristics

A final criteria in terms of assessing online sources had to deal with different website characteristics, namely, the layout of the site and whether or not the site has been properly updated. In terms of website layout, Angela discussed how she checks to see if a site has a professional look and feel, and based on that criteria, is able to better judge to see if the information is legitimate and credible, as stated here: “For me, first off the way it looks…like whoever put the website together probably knows what they’re talking about. It’s not just some HTML page that doesn’t have graphics or anything, like it looks good.”

For Kyle and Diego, an important consideration is when the website has last been updated with the most recent information. Diego claimed, “You really have to make sure the information is up to date and that it’s a legitimate source. I’m sure there are multiple ways to deal with that…make sure that whatever you do has been confirmed to work.” According to Kyle, an updated site is very important, as the medical field is constantly changing and evolving:

The problem is knowing which sources are trustworthy, and out of those sources, which has the most credible information. Because the site may have not been updated since 20 years ago, and we have so many advances in medicine. Knowing what's trustworthy, and then out of those, which are the most updated and comprehensive information.

###  RQ2: How Do Young Adults Make Meaning of Online Health Information Seeking in General?

#### Accessibility

Participants were favorable toward the online space for health information because of it’s ease, convenience, and accessibility of information, as Jack said, “You can try to look up almost anything on the Internet and find almost anything on the Internet.” Max agreed, stating, “It’s always there. It’s information you don’t have to make an appointment with somebody or call somebody. It’s always at your hands, it’s on your phone, there’s live talks you can access when you need health information, and you can go to WebMD.” Matt was a huge fan of using the Internet to find just about any kind of health information he needs or wants:

It's an incredible plethora of knowledge right at your fingertips. Decades and decades and hundreds and hundreds of research and information at your fingertips. You can find anything. If I'm suffering from a serious illness it's not a replacement for going to the hospital or making regular appointments, but it's such a vital tool to help improve your general knowledge of health. Helpful practices, helpful tips, helpful things to do to better yourself mentally and physically.

#### Time Saver

The participants felt that going online for health information is a huge time saver that allows you to get information quickly, without needing to make an appointment with their doctor, or to gauge symptoms while waiting to see a doctor. As Mandy mentioned:

The availability, and you can access it quick. You don’t have to call and wait for your doctor to answer an email or wait a couple of days. If you want to go to a reliable website, go to WebMD or something. If you have your information right there, you can search it and learn more about it. It’s just so quick and readily available, it’s so important.

Angela had similar thoughts, and said, “I think it’s really important because a lot of time you don’t have the ability to just [be] like, ‘oh let me call my doctor.’ You can’t just do that just with ease without having to get an appointment or having to talk to a nurse practitioner or somebody that’s not really your doctor.”

#### Verifies Visits to the Doctor

Participants claimed that using the Internet for health information is helpful because it arms them with information that they can take to their doctor, which in turn they can use as a second opinion or to cross-reference what they are told by their doctor. As Angela stated, “I’ve had concerns about sicknesses or whatever and then I’ll get information from my doctor and then I’ll go to the Internet and a lot of the same things she says will come up.” Elizabeth talked about this notion as a pro of being able to search online for health information, and said:

Well, searching online…a pro would be just getting a general idea of what you’re dealing with and what you think you have so that you can take that information to the doctor because ultimately if you do have something you should go to the doctor. I think that’s the benefit of searching…just having a general idea and better being able to explain it to your physician.

#### Recommendations to Incorporate Social Media

The participants offered some suggestions in terms of how social media can be used in terms of health campaigns and messaging. Some participants gave specific examples by channel, such as Facebook or Twitter, while others had more general ideas of how social media can be used. For example, Kyle had some suggestions in terms of using Facebook:

I think Facebook…if there was a group for a certain condition, a Facebook campaign would be extremely helpful. Just using Facebook as word of mouth among friends. If it's important. If you go to someone with friends, targeting certain conditions, that would be helpful. I would think Facebook would be a valuable tool.

Diego warned about people’s perceptions of Facebook, claiming that some may not view its content to be valid and true. However, he also explained that it could be a useful tool if it were backed by credible health organizations:

I think if health organizations would become more integrated with it and would actually become more supportive, like if they contacted the social media providers directly and create their own source of information and actually tried to use it to their advantage…not haphazardly put it together then they can make sure the health information being spread is accurate and not complete falsehoods.

For Matt, he sees the following of credible health sources similar to following other well-known personalities for any type of information, and that social media can be used as a way to share knowledge already being disseminated via other traditional forms of media:

…there are several effective ways it can be there. Functions where you're able to follow accredited associations, industry personnel, celebrities in the industry… I'm sure someone like Dr. Oz or other respected medical professionals, where they are regionally or nationally known are able to provide various information on their respected pages. If they want to link to published studies, or different health risks that have spread recently. I would think if social media were to be the most effective as possible it would need to be piggybacked by people of that level, people who have an influence, people with media experience, not just social media but media generally. So they can use their following and spring board it to social media to broaden their reach.

Similar to a Google Med site, Nancy offered a suggestion in terms of what features this type of channel could provide:

I would say maybe like have some type of question answer… a place where someone could go in and type a specific symptom or a question or anything that they have about any type of sexually transmitted disease or anything and have some type of way they can get real answers directed towards their question, not just general information.

#### Wariness About Using Social Media for Health

However, in spite of the potential opportunities social media can offer to the participants in terms of their health, there were those who were a bit more cautious and wary about using the channel for health purposes. As Tina put it:

I don't think it can be used in a credible way. A lot of social media…people don't post the right information. People mostly use it for their personal life, I don't think it's informational, it's more recreational, so I wouldn't go on there to get health information.

Rose agreed, and said, “In a way there's that barrier of what's personal and what's not. I don't think a lot of people are gonna talk about their body and what they're going through online. I don't think it would be very helpful.”

Elizabeth felt similarly to Tina and Rose, and felt that social media is too casual a channel to be sharing personal health information:

Yeah, I don’t think its the best avenue to take right now like I said unless you really know the doctor personally…maybe he has a large enough portfolio that he would have a decent amount of followers to check out what he’s putting out there. Otherwise I think it’d be hard for people to trust that, it just seems a bit too casual I think, which is why I think you would need to know them.

## Discussion

### Preliminary Findings

This study explored how young adults made meaning of online health information seeking in general, and what strategies they employed to assess the quality of online sources. The participants shared several benefits to this mode of health information seeking, claiming that it made for more productive visits with doctors and made health information more readily accessible through a variety of different formats. Additionally the participants demonstrated their eHealth literacy competencies by discussing how they assessed online health information, engaging in a series of strategies that encompassed different aspects of eHealth literacy.

### Benefits and Opportunities

Since the majority of young adults are comfortable with using computers and surfing the Internet, participants seemed fairly confident in their competency to find the health information they needed whenever it was needed [35]. Many of the participants embraced the immediacy of online information, which allows them to gather health information at their convenience in a more self-guided manner [36]. Furthermore, participants are given so many options in terms of how to gather content, choosing from many different channels including laptops, desktops, mobile phones, or tablet devices [36]. The challenge for health communicators and campaign developers, as was mentioned by one participant, is determining where people are most frequently turning to and disseminating messages that will attract their attention amongst the online clutter.

Once that information is found and processed, however, participants discussed the benefit of bringing that information to their visits with a doctor. Wald et al [37] argued that this helps create more efficient use of clinical time, and participants claimed that it helped them to better understand their doctor’s prognosis. This also increases the potential for more shared decision-making between doctors and patients, shifting doctor-patient interactions toward a more collaborative communication model, where patients are seen as peers who openly discuss health options and make mutually satisfying decisions [38,39]. This involves teamwork, effective communication, and critical listening on both sides of the interaction, where both doctors and patients can ask clarifying questions and work together to develop and meet shared goals [40].

Social media channels were brought up by the participants as relatively new tools that can be used to assist in the seeking, understanding, and sharing of health information. According to Chou et al [41], social media for health can be beneficial for several reasons: (1) social media can increase perceived social support and interconnectivity among individuals; (2) information sharing is more democratic and patient controlled; and (3) public health programs have recently demonstrated success utilizing social media for health promotion efforts such as smoking cessation and dietary interventions. Furthermore, because social media content is so easy to repost and share with others, there is an inherent viral nature to using these tools, relying on word of mouth and the social context the message is embedded in to ultimately persuade individuals to change their attitudes or behavior surrounding a particular health topic or condition [42,43]. Thus, social media provide a unique opportunity for health communicators and health consumers to develop online communities, spark health discussion, and engage in real-time interactions [26], as was seen as a great benefit to participants. However, participants also cautioned about the use of social media in regards to its informal nature, warning users to evaluate sources accordingly and to use these channels as supplementary outlets of information for more traditional channels [25].

### Assessment and eHealth Literacy

The participants discussed how they assessed the quality of online health information sources, which demonstrated how they utilized different components of eHealth literacy as defined by Norman and Skinner [12]. Participants utilized their cognitive and critical thinking skills to use appropriate searching strategies (media literacy), locate relevant information (information literacy), read and shift through information (traditional literacy), filter the most useful nuggets (information literacy) and overall evaluate the usefulness of online health information (media literacy) by way of tapping into their levels of the analytic types of literacy as mentioned by Norman and Skinner [12]. Furthermore, obtaining access to computers and current information technology (computer literacy), understanding the science behind health information via cross-checking sites (scientific literacy), and being able to harness information that toward making sound health decisions (health literacy) were additional ways participants utilized the context-specific components of eHealth literacy that assisted participants in their assessment of online health information. Therefore, the findings of this study showed that the participants indeed used all aspects of eHealth literacy when it came to searching for sexual health information online.

### Limitations and Future Research

Although qualitative methods allowed for an in-depth understanding of sexual online health information seeking and evaluation through the lens of eHealth, eHealth literacy, and social media, a major limitation is the actual topic of study. Because sexual preferences, activity, and STDs are very intimate and personal experiences, participants may not have been as comfortable responding as honestly as they would with a close friend [44] - in particular, participants may have been hesitant to openly discuss their sexual health matters, or they may have inflated their experiences to make them sound more sexually experienced than they actually are, which happens most frequently with men [45]. In addition, another limitation is that college students may not be totally representative of all online health information seekers. Although they do comprise a majority of the young adult population, which was the population of interest for this study, future studies could explore this phenomenon with additional populations to determine whether or not there are similarities or differences with the findings. In a similar vein, interviews have been critiqued for being contrived and unnaturalistic, occurring in an artificial setting developed by the researcher [46]. Future research could also explore other aspects of sexual health not covered in this study, such as healthy relationships, additional forms of contraception, and negotiating open communication between partners about topics such as STD history or birth control. Future work could also further explore the difference between young adults in the US vs. other countries, as the social and political context within different areas could play a role in online information seeking behaviors, especially sexual health.

### Conclusion

The use of the Internet and technology for health purposes is a growing area for both scholarship and practice that has strong implications for health consumers, medical professionals, and communicators alike. Because the realm of eHealth is relatively new, there are great opportunities to explore this phenomenon through in-depth research, which was the major goal of this study. Specifically, this research explored how young adults made meaning of online health information via eHealth and eHealth literacy. The findings that emerged through 50 in-depth interviews with young adults demonstrated that the online space is an acceptable channel through which they can find and share information. However, in spite of the almost universal usage of social media by this particular group, the findings showed that young adults were hesitant and wary of the channel, not seeing it as a resource for health information but more of a channel for networking and entertainment. In spite of this, this study shows that the online health information seeking behaviors of young adults is an area that warrants further exploration. As the number of individuals on the Web continues to increase, so does the need for more research on online health information.
